# Divergent Effects of HSP70 Overexpression in Photoreceptors During Inherited Retinal Degeneration

**DOI:** 10.1167/iovs.61.12.25

**Published:** 2020-10-27

**Authors:** Ke Jiang, Elizabeth Fairless, Atsuhiro Kanda, Norimoto Gotoh, Tiziana Cogliati, Tiansen Li, Anand Swaroop

**Affiliations:** 1 Neurobiology, Neurodegeneration, and Repair Laboratory (NNRL), National Eye Institute, National Institutes of Health, Bethesda, Maryland, United States

**Keywords:** photoreceptors, retinal degeneration, heat shock protein, proteostasis, chaperone

## Abstract

**Purpose:**

Disruption of proteostasis is a key event in many neurodegenerative diseases. Heat shock proteins (HSPs) participate in multiple functions associated with intracellular transport and proteostasis. We evaluated the effect of augmented HSP70 expression in mutant photoreceptors of mouse retinal degeneration models to test the hypothesis that failure to sustain HSP70 expression contributes to photoreceptor cell death.

**Methods:**

We examined HSP70 expression in retinas of wild-type and mutant mice by RNA and protein analysis. A transgenic mouse line, *TgCrx-Hspa1a-Flag*, was generated to express FLAG-tagged full-length HSP70 protein under control of a 2.3 kb mouse *Crx* promoter. This line was crossed to three distinct retinal degeneration mouse models. Retinal structure and function were evaluated by histology, immunohistochemistry, and electroretinography.

**Results:**

In seven different mouse models of retinal degeneration, we detected transient elevation of endogenous HSP70 expression at early stages, followed by a dramatic reduction as cell death ensues, suggesting an initial adaptive response to cellular stress. Augmented expression of HSP70 in *RHOT17M* mice, in which mutant rhodopsin is misfolded, marginally improved photoreceptor survival, whereas elevated HSP70 led to more severe retinal degeneration in *rd10* mutants that produce a partially functional PDE6B. In *Rpgrip1^−^^/^^−^* mice that display a ciliary defect, higher HSP70 had no impact on photoreceptor survival or function.

**Conclusions:**

HSP70 overexpression has divergent effects in photoreceptors determined, at least in part, by the nature of the mutant protein each model carries. Additional investigations on HSP pathways and associated chaperone networks in photoreceptors are needed before designing therapeutic strategies targeting proteostasis.

Inherited retinal degenerative diseases (IRDs), characterized by progressive dysfunction or loss of photoreceptors, constitute a major cause of vision impairment, which severely impacts patients’ quality of life. Although much progress has been achieved with mutations identified in over 200 genes,^1^ we lack a precise understanding of pathogenic mechanisms and effective treatments for most IRDs. Proteostasis, defined as the appropriate production, folding, trafficking, and degradation of proteins, is essential for cell's health and survival. Given the highly active phototransduction cascade and continuous renewal of outer segments (OS), maintenance of proteostasis is critical for healthy photoreceptors and for the survival of stressed photoreceptors in IRDs. Disrupted cellular proteostasis and resulting apoptosis driven by the unfolded protein response (UPR) are among several hypotheses to explain the mechanism underlying photoreceptor death in degenerating retina.^2^^–^^5^

Molecular chaperones, especially the heat shock protein (HSP) family, act to support cellular proteostasis by restoring protein function under physiological conditions or by triggering programed cell death under stress.^6^^,^^7^ The HSP superfamily is classified by apparent molecular mass into subfamilies. Eight HSP70 proteins identified in humans, belonging to the 70-kDa subfamily, share 52% to 99% amino acid sequence homology and exhibit similar functions yet somewhat distinct characteristics.^8^ Hereafter we use the term “HSP70” to collectively refer to two proteins, HSP70-1a and HSP70-1b, which only differ from each other by two amino acid residues and are encoded in mice by *Hspa1a* and *Hspa1b* genes, respectively.^8^^,^^9^ HSP70 is one of the most studied chaperones, especially in the stressed retina. In addition to the stress-inducible HSP70, a cognate 70-kDa HSP HSC70 (*Hspa8*)^10^ is constitutively expressed. Under physiological conditions, HSPs protect and improve folding of newly synthesized proteins, participate in quality control, guide their translocation into distinct organelle destinations, and assist in the assembly/disassembly of multiprotein complexes.^11^^–^^13^ When under stress, transcription regulator heat shock factor 1 (HSF1) activates expression of various HSPs, including HSP70.^14^ HSP70 binds to misfolded proteins and facilitates their recovery with assistance of other HSPs, such as HSP40 and HSP90, and co-chaperones such as Hip and Hop.^15^^,^^16^ Alternatively, when the damage is not repairable, HSP70 facilitates the degradation of aggregated proteins by delivering them to the ubiquitin proteasome system with assistance of co-chaperone CHIP.^17^^,^^18^

The nexus between disruption in cellular proteostasis and progression of neurodegenerative disease has uncovered an important role for chaperone proteins in regulating the survival or death of stressed neuronal cells.^19^^–^^21^ Disease-causing proteins, such as β-amyloid and tau in Alzheimer disease, huntingtin in Huntington disease, α-synuclein in Parkinson disease, and mutant rhodopsin in retinitis pigmentosa, all interact with the HSP chaperone machinery.^20^^–^^22^ Therefore HSPs are potential therapeutic targets for neurodegenerative diseases.

Although HSP70 deficient mice were generated over a decade ago, the role of HSP70 in retinal development has not been investigated.^23^ In the adult retina, HSP70 maintains proteostasis and plays an essential role in the maturation of phototransduction proteins.^24^^,^^25^ In cooperation with HSP90 and a photoreceptor specific co-chaperone AIPL1, HSP70 is implicated in facilitating correct folding and assembly of retinal cGMP phosphodiesterase (PDE6) holoenzyme.^26^^–^^28^ Elevated HSP70 expression was observed in photoreceptors following induced retinal detachment and hypothesized to play a protective role.^29^ HSP70 upregulation was also reported to delay *N*-methyl-*N*-nitrosourea-induced photoreceptor death.^30^ Furthermore, inhibition of HSP90, a negative regulator of HSF1 activation and of HSP70 expression, leads to improved photoreceptor survival and visual function in a rat model with rhodopsin P23H mutation.^31^ Exogenous expression of HSP70 and HSF1 are also suggested to significantly improve the survival of injured retinal ganglion cells.^32^^,^^33^ Along these lines, HSP70 is suggested to play a protective role in the retina, especially in stressed photoreceptors, in response to either exogenous stress stimuli or inherited mutations.^29^^–^^32^^,^^34^

Here we tested the hypotheses that compromised HSP70 expression precedes photoreceptor death in IRDs and that expression of exogenous HSP70 could extend the survival of mutant photoreceptors. We first assessed the expression of HSP70 transcripts in RNA-seq data from rods and S-cone-like photoreceptors^35^ compared with whole retina^36^ and then examined HSP70 protein levels in several retinal degeneration mouse models. We augmented HSP70 expression in the photoreceptors of *RHOT17M*, *Rpgrip1^−^^/^^−^*, and *rd10* mice by crossing them with a transgenic mouse line, *TgCrx-Hspa1a-Flag*, that overexpresses HSP70. Our studies demonstrate divergent effects of HSP70 expression in degenerating photoreceptors carrying different mutations and highlight the importance of distinct approaches when targeting cellular proteostasis for therapy.

## Methods

### Mice

Mice were reared under ∼200 lux white, fluorescent cyclic (12 hr/12 hr light/dark cycle) light conditions following recommendations of the Guide for the Care and Use of Laboratory Animals, the Institute of Laboratory Animal Resources, and the Public Health Service Policy on Humane Care and Use of Laboratory Animals. All protocols were approved by the Animal Care and Use Committee of the National Eye Institute (NEI ASP# 650). Animals were fed NIH-31 Open Formula Mouse Diet (7017) (ENVIGO, Frederick, MD, USA). The following retinal degeneration mouse lines were used: *TgRHOT17M*^37^ (referred to as *RHOT17M*), *Rpgrip1^tm1Tili^*^38^ (*Rpgrip1^−^^/^^−^*), and *Pde6b^rd10/rd10^* (*rd10*) from Jackson Laboratory (Bar Harbor, ME, USA). Other mouse models include: C57BL/6 (wild-type), *Pde6b^rd1/rd1^*(*rd1*), *Cep290^rd16/rd16^*(*rd16*),^39^ *Prph2^rd2/rd2^*(*rds*) (Jackson Laboratory), and *Nrl^−^^/^^−^;Nrl-GFP* (*Nrl^−^^/^^−^*).^40^ Both male and female mice were used in this study.

### Generation of TgCrxp-Hspa1a-Flag Mice

A 2.3 kb mouse *cone-rod homeobox (Crx)* promoter (from −2286 to +72) and the *Hspa1a*-coding region with an additional Kozak sequence and FLAG-tag were cloned into a modified promoter-less pCl vector (Promega, Madison, WI, USA). The *Crx:Hspa1a-Flag* fragment was purified and injected into fertilized FVB/N mouse oocytes. Transgenic founder mice and their progeny were identified by PCR. The founders were backcrossed to *C57BL/6J* mice to establish a stable colony. *TgCrxp-Hspa1a-Flag* mice are referred to as *TgHspa1a+,* whereas littermate controls without the transgene are called *TgHspa1a-.*

### Histology of Retinal Cross-Sections

Eyes were enucleated and marked on dorsal side. Eyeballs were fixed in 4% glutaraldehyde followed by 4% paraformaldehyde, embedded in methacrylate and sectioned at 1-µm thickness along the dorsal-ventral axis. Pictures of hematoxylin and eosin (H&E)-stained retinal sections were taken at 20X and 40X magnifications. Outer nuclear layer (ONL) thickness was determined from sections displaying optic nerve head. Only the dorsal half was measured by taking nine measurements at equal spacing from the optic nerve head to the periphery on each retinal section.

### Immunoblotting and Immunofluorescence Microscopy

Whole retina was homogenized in radioimmunoprecipitation assay buffer. Retinal proteins were separated by SDS-PAGE and transferred to polyvinylidene difluoride membranes. Membranes were blocked with StartingBlock (Thermo Fisher, Rockford, IL, USA) for 1 hour at room temperature and incubated overnight at 4°C with the primary antibody. The following primary antibodies were used: mouse anti-HSP70 (1:1000, Cat. #ADI-SPA-810; Enzo, Farmingdale, NY, USA), rabbit anti-FLAG (1:3000, Cat. #2368; Cell Signaling, Danvers, MA, USA), and mouse anti-β-actin (1:2000, Cat. #MABT825; Millipore Sigma, St. Louis, MO, USA). Blots were washed three times using Tris-buffered saline with Tween-20 (137 mM sodium chloride, 20 mM Tris, 0.1% Tween-20, pH 7.6) and incubated with horseradish peroxidase-conjugated donkey anti-rabbit or anti-mouse secondary antibody (Jackson Immunoresearch, West Grove, PA, USA) for 1 hour at room temperature. The membranes were developed using SuperSignal West Pico or Femto Chemiluminescent substrate (Thermo Fisher).

For immunofluorescence, eyes were enucleated and fixed in 4% paraformaldehyde for 30 minutes, cryo-preserved through a series of sucrose gradients, cryo-sectioned at 10-µm thickness, and processed as previously described.^41^ Primary antibodies were used at the following dilutions: rabbit anti-FLAG (1:600, Cat. #2368; Cell Signaling), mouse anti-HSP70 (1:200, Cat. #ADI-SPA-810; Enzo), mouse anti-rhodopsin (1:600, Cat. #MAB5356; Millipore Sigma), rabbit anti-calreticulin (1:400, Cat. #12238; Cell Signaling), and rabbit anti-PDE6B (1:200; Dr. Tiansen Li). Secondary antibodies conjugated with Alexa Fluor 488 or 594 (Thermo Fisher) were used at a dilution of 1:1000. Nuclei were counterstained with 4′,6-diamidino-2-phenylindole. Images were acquired using a Zeiss 700 confocal microscope (Carl Zeiss Meditec, Dublin, CA, USA).

### Electroretinography (ERG)

ERG recordings were performed with a computer-based system (E2, Diagnosys LLC, Lowell, MA, USA), using flashes produced with LEDs or Xenon bulbs. Mice were dark-adapted overnight and anesthetized with an intraperitoneal injection of ketamine (80 mg/Kg) and xylazine (8 mg/Kg). Pupils were dilated with topical phenylephrine (2.5%) and tropicamide (0.5%). Corneal ERGs were recorded from both eyes using gold wire loop electrodes with a drop of 2.5% hypromellose ophthalmic demulcent solution. Reference was taken with a gold wire loop placed in the mouth. For dark-adapted ERG, mice were stimulated with flashes of increasing light intensity (from 0.0001–1000 cd.s.m^−2^). Responses were computer-averaged and recorded at 3 to 60 second intervals depending on the stimulus intensity. For light-adapted ERG, mice were light-adapted for 2 minutes and were stimulated with flashes (from 0.3–100 cd.s.m^−2^) in the presence of a white 32 cd.m^−2^ rod-suppressing background.

## Results

### HSP70 Expression in the Developing and Degenerating Retina

**Figure 1.**
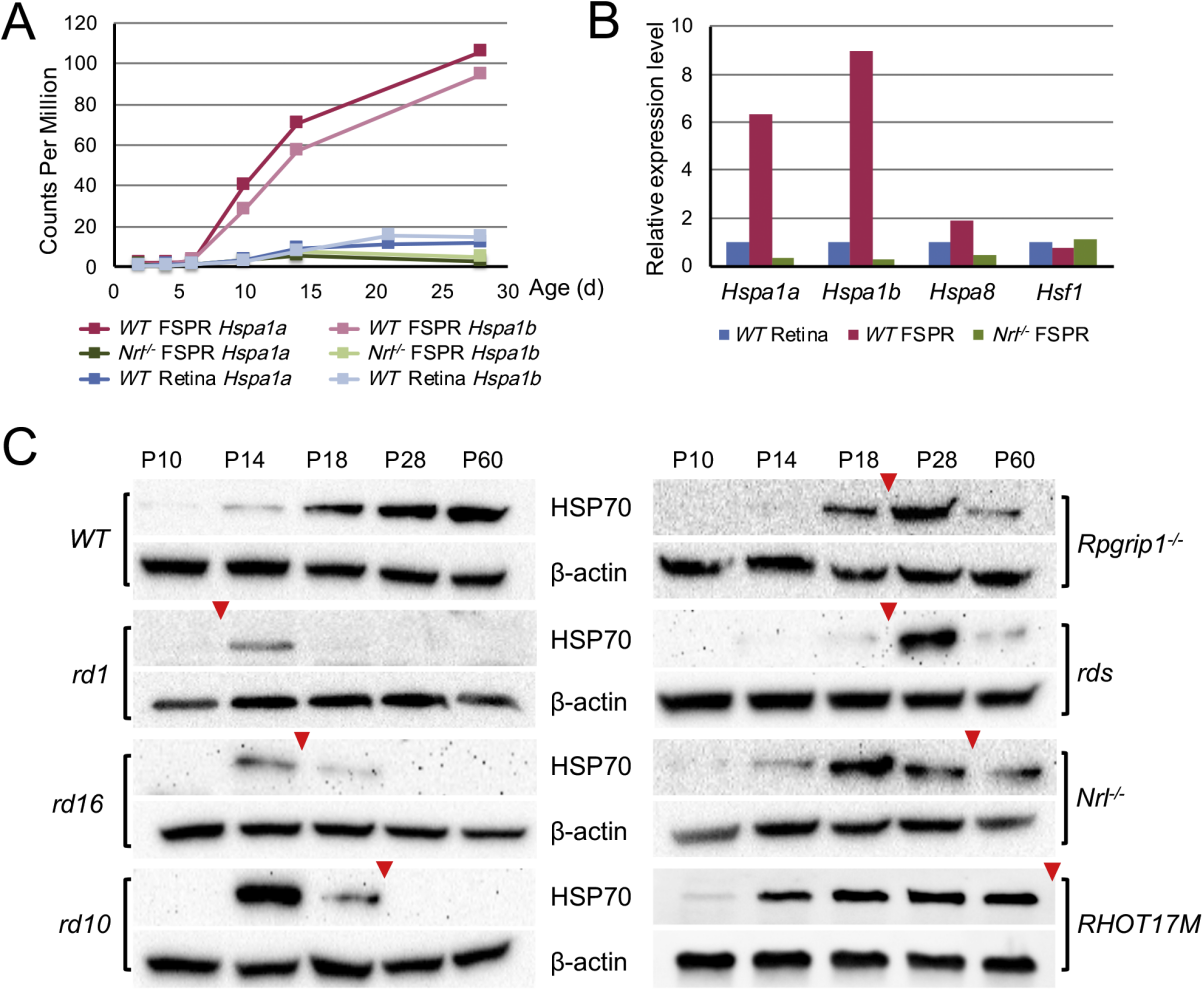
HSP70 expression in developing and degenerating retina. (**A**) Expression level of the two transcripts of HSP70, *Hspa1a* and *Hspa1b,* during retina/photoreceptor maturation from P2 to P28, determined by RNAseq in flow sorted photoreceptors (FSPR) from *Nrl-GFP* mice (*WT* FSPR) or *Nrl^−^^/^^−^* mice (*Nrl^−^^/^^−^* FSPR) and in whole *Nrl-GFP* (*WT*) retinas. Each data point represents the average of two measurements (*n* = 2). (**B**) Relative expression levels of *Hspa1a* and *Hspa1b* at P28 in FSPR from *Nrl-GFP* mice or *Nrl^−^^/^^−^* mice compared with that in whole *Nrl-GFP* retinas. Also shown are relative expression levels of two other HSP family related genes *Hspa8* and *Hsf1*. (**C**) Protein level of HSP70 in whole retinas of wild-type (*Nrl-GFP*) and seven retinal degeneration models, *rd1*, *rd16*, *rd10*, *Rpgrip1^−^^/^^−^ rds*, *Nrl^−^^/^^−^*, and *RHOT17M*, at a series of ages from P10 to 2 months. *Arrowheads* indicate the time point in each model when approximately 50% of photoreceptors are still alive.

We previously reported developmental transcriptome profiles of rods (flow sorted from *Nrl-GFP* mice) and S-cone-like photoreceptors (flow sorted from *Nrl^−^^/^^−^;Nrl-GFP* mice) and of whole retinas.^35^^,^^36^^,^^42^ Focusing on chaperone genes, we noticed that expression of the two HSP70 transcripts, *Hspa1a* and *Hspa1b,* in rods progressively increased from postnatal day 2 (P2) to P28 (Fig. 1A). Compared with an even distribution of the transcription factor *Hsf1*, expression of the cognate *Hspa8* was somewhat enriched in rods at P28 (Fig. 1B)*.* Notably*, Hspa1a* and *Hspa1b* showed dramatically higher expression in rods at P28 compared with S-cone-like photoreceptors and the whole retina (Fig. 1B). Accordingly, HSP70 protein increased in temporal correlation with maturation of the retina (Fig. 1C). Immunoblotting of retinal extracts from seven mouse models (*rd1*, *rd16*, *rd10*, *Rpgrip1^−^^/^^−^*, *rds*, *Nrl^−^^/^^−^*, and *RHOT17M*) revealed transient expression of HSP70 at early/mid stage of degeneration, except in the *RHOT17M* retina (Fig. 1C). The timing of HSP70 expression correlated with the severity of disease. In mouse models with a relatively fast degeneration (*rd1*, *rd16*, and *rd10*), HSP70 expression was observed at P14, whereas relatively slow mouse models (*Rpgrip1^−^^/^^−^*, *rds*, and *Nrl^−^^/^^−^*) revealed later expression from P18 to P28. We also noted that HSP70 expression dramatically decreased during the progression of photoreceptor degeneration in the earlier mentioned six models. Distinctively, HSP70 expression was evident at P14 in a slow degeneration model *RHOT17M* and persisted as the degeneration progressed. In contrast, we observed a stable temporal expression of the constitutively expressed cognate HSC70 and HSP90, a member of the 90-kDa subfamily, in degenerating retinas (data not shown).

### Generation and Characterization of TgCrx-Hspa1a-Flag Mice

**Figure 2.**
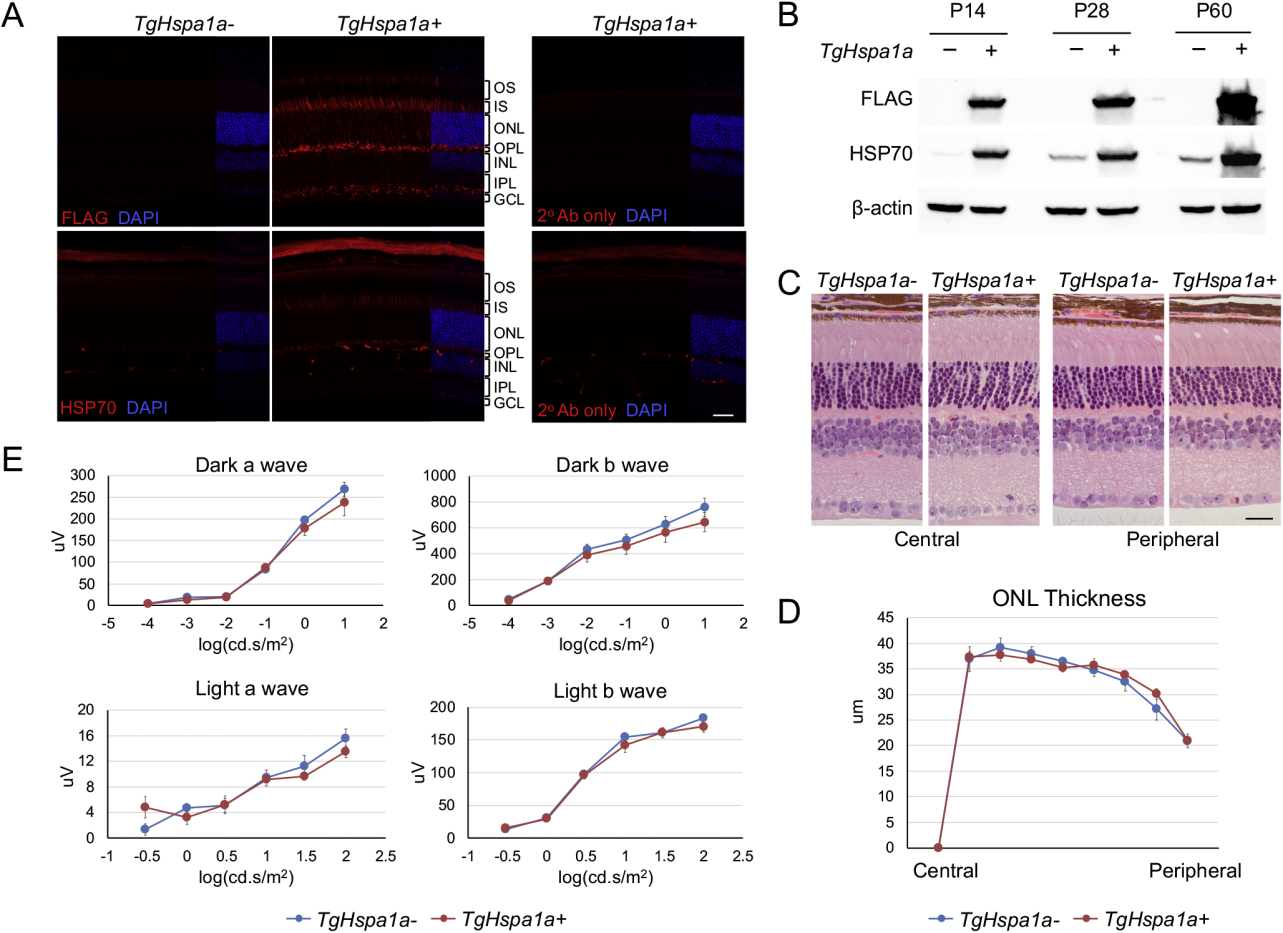
Augmented expression of HSP70 from *TgCrx-Hspa1a-Flag* transgene in wild-type retina. (**A**) Immunofluorescent labeling of FLAG and HSP70 in retinal cross-sections from *TgHspa1a+* mice and *TgHspa1a-* littermates at P28. (**B**) Immunoblots showing elevated expression of HSP70 protein driven by the *TgCrx-Hspa1a-Flag* transgene at P14, P28, and P60. (**C**) Representative picture of H&E stained retinal cross-sections, and (**D**) plot of ONL thickness measurements from *TgHspa1a+* (*n* = 4) and *TgHspa1a-* littermates (*n* = 5) at 8 months. (**E**) ERG response in *TgHspa1a+* (*n* = 3) and *TgHspa1a-* littermates (*n* = 6) at 8 months. *Scale bar* for all panels: 25 μm. *Error bar*: SEM. OS, outer segment; IS, inner segment; ONL, outer nuclear layer; OPL, outer plexiform layer; INL, inner nuclear layer; IPL, inner plexiform layer; GCL, ganglion cell layer.

To test the potential neuroprotective effect of HSP70, we generated a transgenic mouse line with augmented HSP70 expression in both rod and cone photoreceptors, controlled by a 2.3 kb mouse *Crx* promoter. The *TgCrx-Hspa1a-Flag* (referred to as *TgHspa1a+*) mouse expresses a FLAG-tagged full-length HSP70 protein. Immunofluorescent labeling of FLAG on *TgHspa1a+* retina revealed transgene expression in photoreceptors with strong signals in the inner segment (IS) and synaptic terminals (Fig. 2A). Weaker staining of FLAG was observed in inner plexiform layer, possibly resulting from *Crx* promoter activity in bipolar cells^43^ (Fig. 2A). Robust expression of HSP70 protein driven by the transgene was detected by immunoblotting in P14 *TgHspa1a+* retinas, and the expression gradually increased at P28 and P60 (Fig. 2B). Eight-month old *TgHspa1a+* mice displayed normal retinal morphology (Fig. 2C) with no difference in ONL thickness between *TgHspa1a+* (*n* = 4) and *TgHspa1a-* (*n* = 5) littermates (Fig. 2D). ERG response (a- and b-wave amplitude) showed no change between *TgHspa1a+* (*n* = 3) and *TgHspa1a-* (*n* = 6) mice in dark- or light-adapted conditions (Fig. 2E). Thus augmented expression of HSP70 does not alter the development, survival, or function of wild-type photoreceptors.

### Improved Photoreceptor Survival but not ERG Response by Augmented HSP70 Expression in RHOT17M Retina

**Figure 3.**
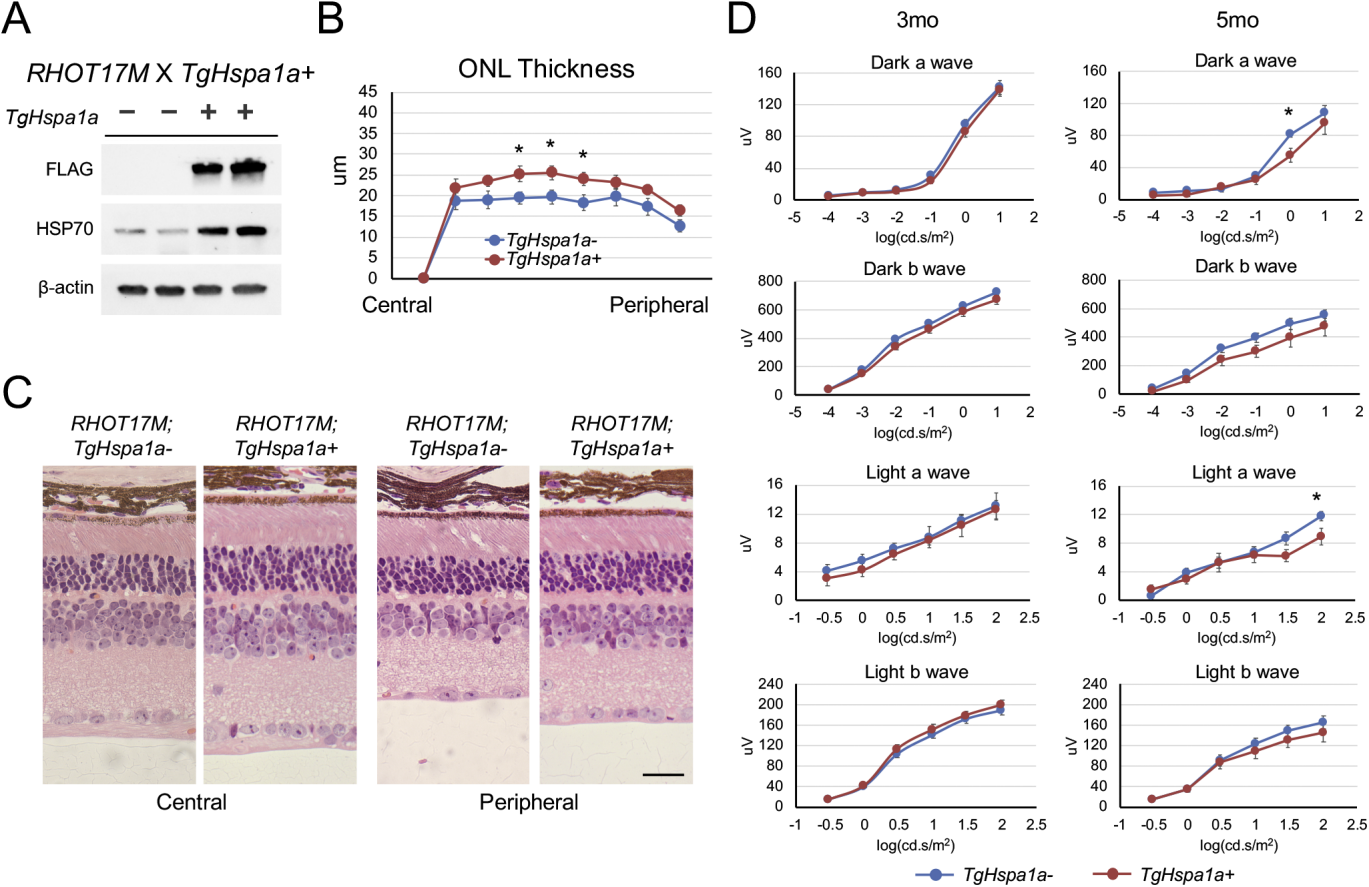
Augmented expression of HSP70 in *RHOT17M* mice. (**A**) Immunoblots confirm the expression of *TgCrx-Hspa1a-Flag* transgene and elevated total HSP70 protein level in *RHOT17M* mice at 3 months of age. (**B**) Marginally increased ONL thickness was observed in *RHOT17M; TgHspa1a+* (*n* = 8) mice at 5 months of age when compared with *RHOT17M; TgHspa1a-* (*n* = 6) littermates. (**C**) Representative pictures of central and peripheral region of H&E stained retinal cross-sections from *RHOT17M;TgHspa1a-* and *RHOT17M;TgHspa1a+* mice at 5 months. (**D**) Plots of scotopic and photopic ERG amplitudes of *RHOT17M; TgHspa1a-* (*n* = 16 and 11, respectively) and *RHOT17M; TgHspa1a+* (*n* = 16 and 9, respectively) mice at 3 and 5 months. *Scale bar*: 25 μm for all panels. *Error bar*: SEM. Student's *t*-test, *P < 0.05.

*TgHspa1a+* mice were crossed to *RHOT17M* mice, which express a class II mutant rhodopsin displaying defects in stability and subcellular localization.^37^^,^^44^ *RHOT17M* mice exhibit slow degeneration with complete photoreceptor loss by 8 months of age.^37^ Immunoblotting confirmed robust expression of HSP70 from the transgene in 3-month-old *RHOT17M;TgHspa1a+* retinas (Fig. 3A). A marginal but significant increase in ONL thickness was observed from center to periphery in cross-sections of 5-month-old *RHOT17M;TgHspa1a+* (*n* = 8) retina compared with littermate controls (*n* = 6) (Figs. 3B, 3C). An increase in inner nuclear layer and inner plexiform layer thickness was also evident. However, transgene expression in mutant retina did not translate into a significant improvement of scotopic or photopic ERG amplitudes (Fig. 3D).

### No Effect on Photoreceptor Survival but Earlier Impairment of ERG Response by Augmented HSP70 Expression in Rpgrip1^−/−^ Retina

**Figure 4.**
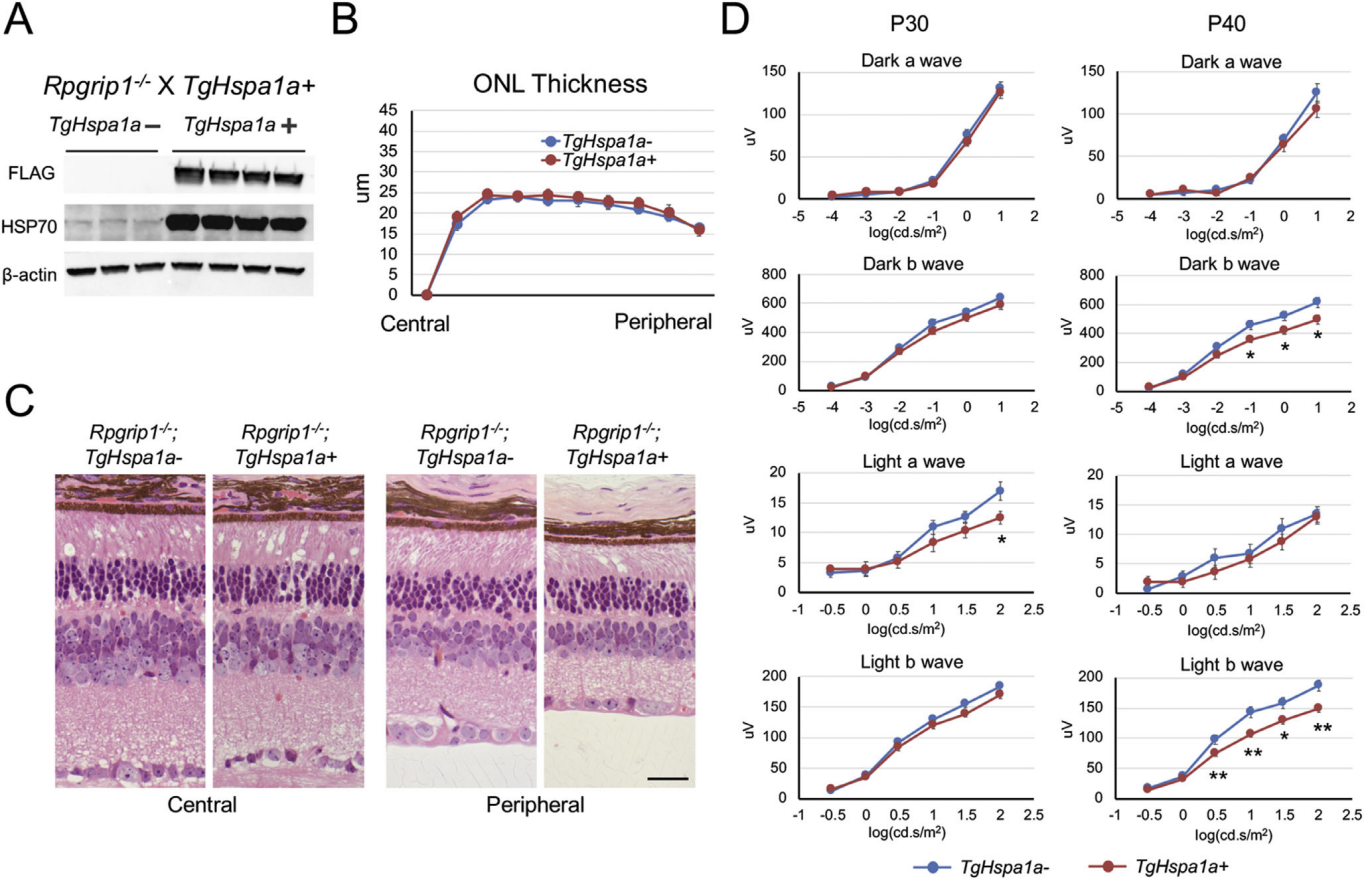
Augmented expression of HSP70 in *Rpgrip1^−^^/^^−^* mice. (**A**) Immunoblots confirm the expression of *TgCrx-Hspa1a-Flag* transgene and elevated total HSP70 protein level in *Rpgrip1^−^^/^^−^* mice at P40. (**B**) ONL thickness is not different between *Rpgrip1^−^^/^^−^; TgHspa1a-* (*n* = 7) and *Rpgrip1^−^^/^^−^; TgHspa1a+* (*n* = 8) mice at P40. (**C**) Representative picture of central and peripheral region of H&E stained retinal cross-sections from *Rpgrip1^−^^/^^−^; TgHspa1a-* and *Rpgrip1^−^^/^^−^; TgHspa1a+* mice at P40. (**D**) Plots of scotopic and photopic ERG amplitudes of *Rpgrip1^−^^/^^−^; TgHspa1a-* (*n* = 14), and *Rpgrip1^−^^/^^−^; TgHspa1a+* (*n* = 18) mice at P30 and P40. *Scale bar*: 25 μm for all panels. *Error bar*: SEM. Student's *t*-test, *P < 0.05, **P < 0.01.

*TgHspa1a+* mice were crossed to *Rpgrip1^−^^/^^−^* mice, which exhibit a ciliary defect with a moderate degeneration rate, showing near complete photoreceptor death at 3 months of age.^38^ Immunoblotting confirmed robust expression of HSP70 from the transgene in P40 *Rpgrip1^−^^/^^−^;TgHspa1a+* retina (Fig. 4A). No difference in ONL thickness was detected between *Rpgrip1^−^^/^^−^;TgHspa1a-* (*n* = 7) and *Rpgrip1^−^^/^^−^;TgHspa1a+* (*n* = 8) retina (Figs. 4B, 4C). Scotopic and photopic ERG amplitudes were not significantly different at P30 (Fig. 4D, left panels) but showed reduction at P40 in mice expressing the transgene (Fig. 4D, right panels).

### Accelerated Photoreceptor Degeneration by Augmented HSP70 Expression in rd10 Retina

**Figure 5.**
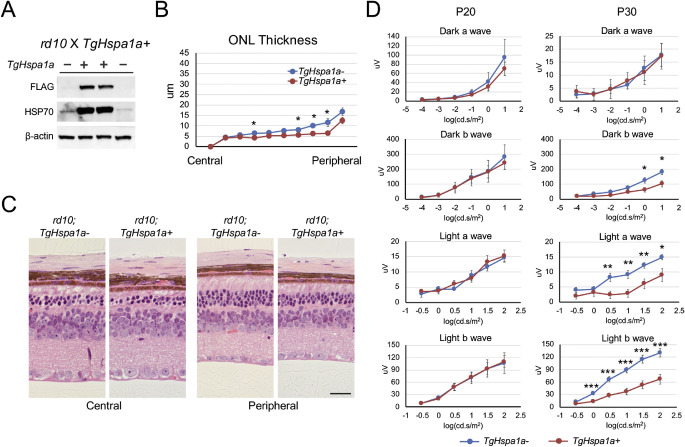
Augmented expression of HSP70 in *rd10* mice. (**A**) Immunoblots confirm the expression of *TgCrx-Hspa1a-Flag* transgene and elevated total HSP70 protein level in *rd10* mice at P20. (**B**) Decreased ONL thickness, more pronounced in the retinal periphery, was observed in *rd10; TgHspa1a+* (*n* = 5) mice at P30 when compared with *rd10; TgHspa1a-* (*n* = 5) littermates. (**C**) Representative picture of central and peripheral region of H&E stained retinal cross-sections from *rd10;TgHspa1a-* and *rd10;TgHspa1a+* mice at P30. (**D**) Plots of scotopic and photopic ERG amplitudes of *rd10; TgHspa1a-* (*n* = 5 and 10) and *rd10; TgHspa1a+* (*n* = 10 for both ages) mice at P20 and P30, respectively. *Scale bar*: 25 μm for all panels. *Error bar*: SEM. Student's *t*-test, *P < 0.05, **P < 0.01, ***P < 0.001.

*TgHspa1a+* mice were crossed to *rd10* mice, which carry a missense mutation in the *Pde6b* gene and display relatively fast degeneration with complete photoreceptor loss at 2 months.^45^ Robust expression of HSP70 from the transgene was detected by immunoblotting in P20 *rd10;TgHspa1a+* retinas (Fig. 5A). More severe degeneration was observed in the peripheral retina of *rd10;TgHspa1a+* (*n* = 5) mice at P30 compared with *rd10;TgHspa1a-* (*n* = 5) littermates (Figs. 5B, 5C). At P30, and not earlier, we observed significantly reduced scotopic b-wave amplitude and dramatically reduced photopic a- and b-wave amplitudes in mice expressing the transgene (Fig. 5D).

### Altered Rhodopsin and PDE6B Expression with Augmented HSP70 Expression in RHOT17M and rd10 Retina

**Figure 6.**
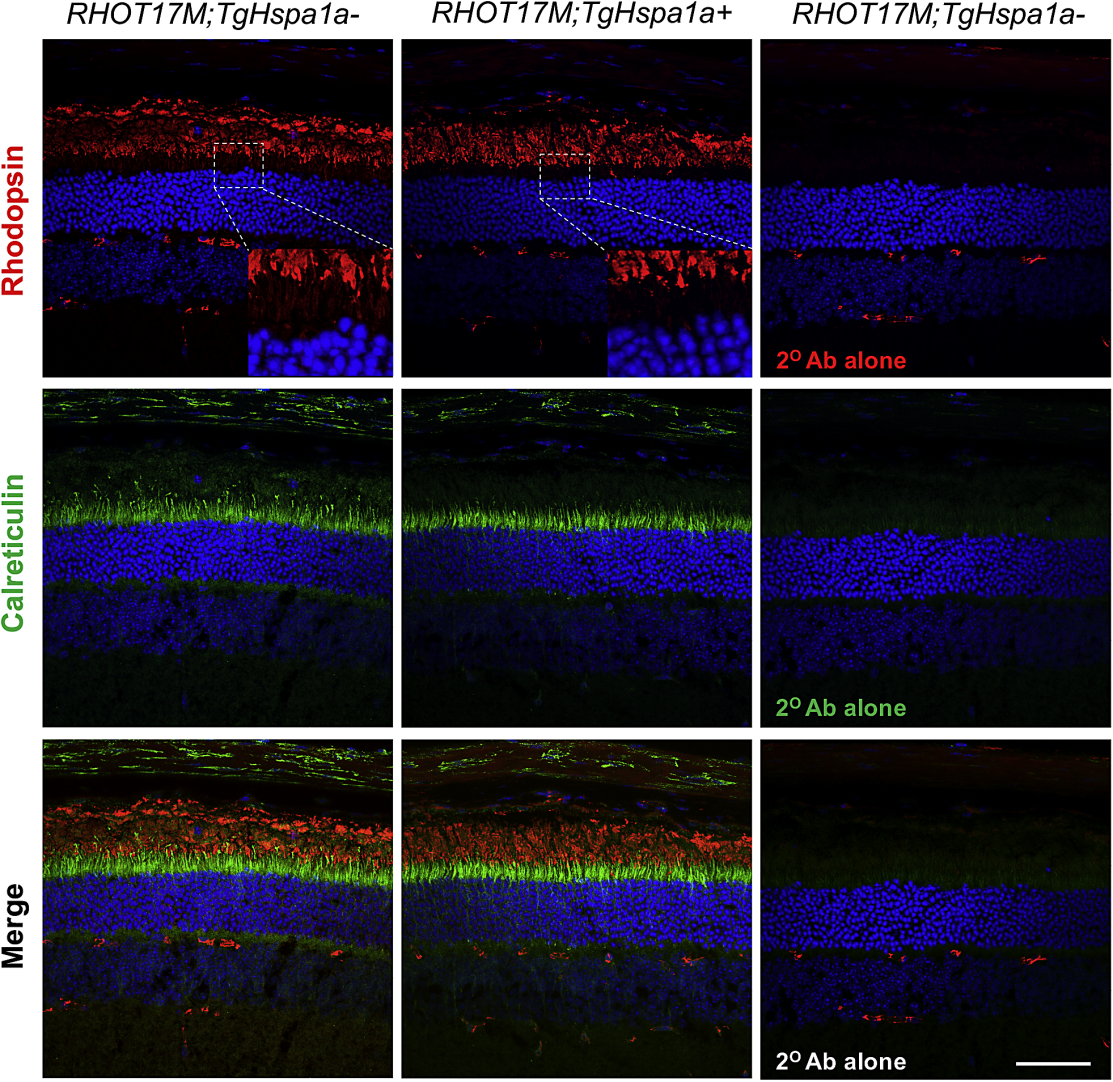
Reduced rhodopsin retention in IS of *RHOT17M; TgHSP70+* retinas. Immunofluorescent labeling of rhodopsin and calreticulin in retinal cross-sections from 3-month-old *RHOT17M; TgHspa1a-* versus *RHOT17M; TgHspa1a+* mice. *Scale bar*: 50 μm for all panels.

**Figure 7.**
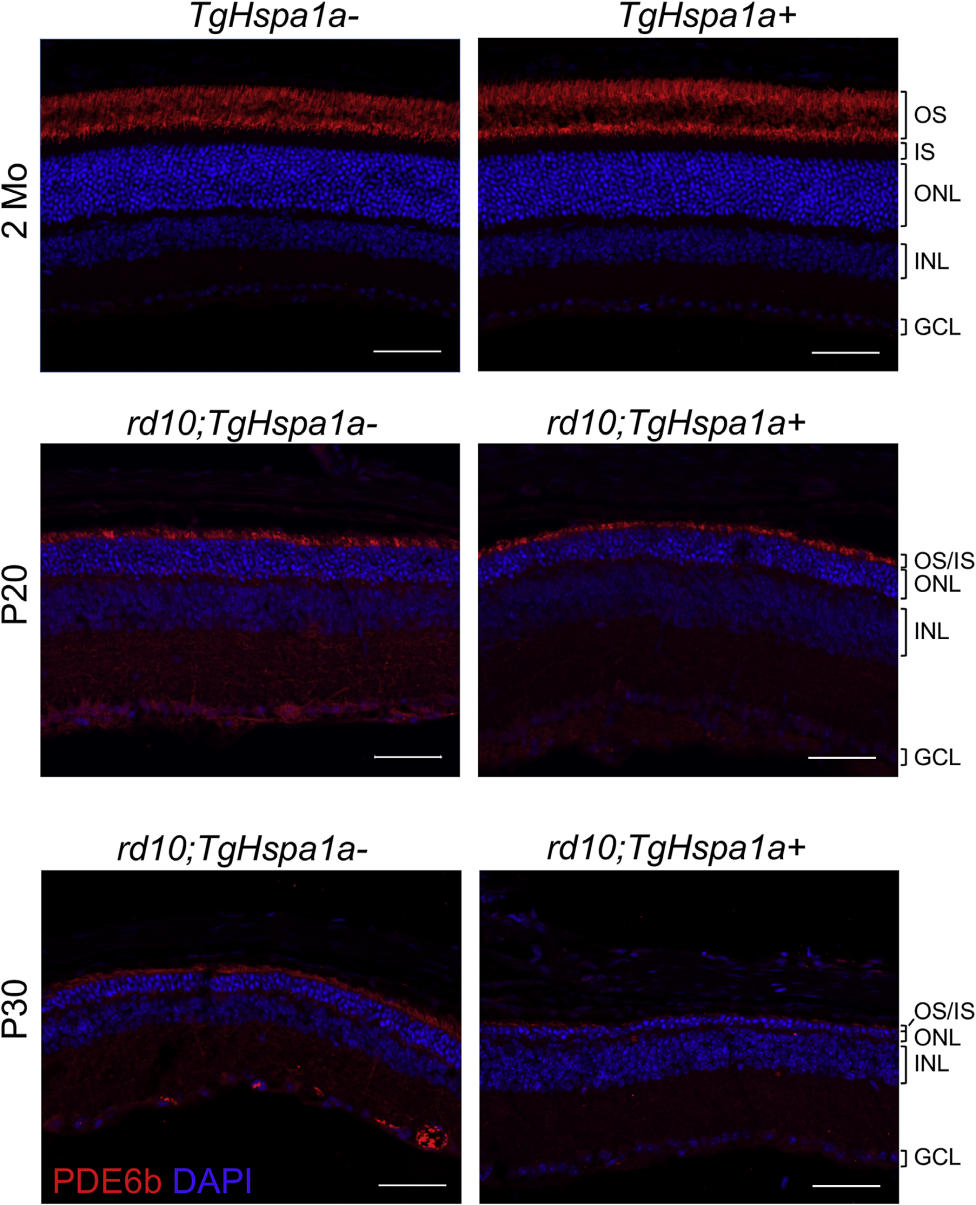
Expression of PDE6B is reduced in *rd10; TgHSP70+* retinas. Immunofluorescent labeling of PDE6b in retinal cross-sections from 2-month-old *TgHspa1a-* versus *TgHspa1a+* mice and *rd10;TgHspa1a-* vs *rd10; TgHspa1a+* mice at P20 and P30. *Scale bar*: 50 μm for all panels. OS, outer segment; IS, inner segment; ONL, outer nuclear layer; INL, inner nuclear layer; GCL, ganglion cell layer.

Immunolabeling revealed robust staining of rhodopsin in the OS of 3-month-old *RHOT17M;TgHspa1a-* and *RHOT17M;TgHspa1a+* retinas (Fig. 6). We observed weaker staining of rhodopsin in the IS area (marked by calreticulin) in *RHOT17M;TgHspa1a+* retina (Fig. 6 insert box), suggesting reduced retention of mutant rhodopsin. High expression of HSP70 in 2-month-old retina under a nondegeneration background (*C57BL/6*) does not affect expression or localization of PDE6B protein (Fig. 7). In the *rd10* retinas, PDE6B expression level at P20 is comparable with or without augmented HSP70 expression, whereas reduced staining of PDE6B was detected in P30 *rd10;TgHspa1a+* mice compared with controls (Fig. 7).

## Discussion

High turnover of proteins makes the photoreceptors especially vulnerable to disruptions of proteostasis, a feature commonly observed in neurodegenerative diseases. Several retinal disease-causing mutations produce misfolded or mistargeted proteins, accumulation of which can trigger cellular responses that eventually lead to demise of the photoreceptors.^46^^,^^47^ Thus manipulation of chaperone system and UPR pathways offers an attractive, potential therapeutic strategy to treat retinal degeneration irrespective of the original mutation. The HSP family of proteins control many aspects of cellular proteostasis including nascent protein folding, trafficking to organelles, repair of damaged proteins, and assembly of multiprotein complexes. In doing so, they promote cellular survival and longevity against proteotoxic stress implying a likely role in neuroprotection; yet there is still no detailed investigation of their therapeutic effects in retinal degenerative conditions.

In this study, we observed transient high expression of HSP70 protein as a common feature in early stages of degenerating retinas. Unexpectedly, augmented HSP70 expression through a transgenic approach manifested divergent effects in the three retinal degeneration mouse models we tested. A protective effect on augmentation of HSP70 expression was only attained in the *RHOT17M* mice. Rhodopsin mutations can be classified into several subgroups based on the mutant protein's structure, stability, function, and intracellular localization.^48^^,^^49^ T17M rhodopsin at low concentration displays altered post-translational modification and reduced stability; but the majority can fold well enough to exit endoplasmic reticulum (ER) and translocate to the OS.^49^ In the *RHOT17M* mice and in transiently transfected cell cultures, T17M rhodopsin manifests reduced thermal stability, resulting in partially misfolded proteins and moderate ER retention, lacking full ability of regeneration with the chromophore 11-*cis*-retinal, thereby failing to integrate in the plasma membrane.^37^^,^^49^^,^^50^ Induction of ER stress was reported in *RHOT17M* retinas,^51^ and manipulating ER stress-related UPR pathway could improve photoreceptor survival in *RHOT17M* mice.^52^^,^^53^ Previous studies examined different methods to improve chaperone activity in treating retinal degeneration related to rhodopsin aggregation.^49^^,^^54^ In this study, improvement in ONL thickness indicated protective effects in the *RHOT17M* retina with augmented HSP70 expression. Reduced retention of mutant rhodopsin in the IS suggests that HSP70 facilitates folding and translocation of mutant rhodopsin to the OS. Further studies are however needed to elucidate the precise mechanism of HSP70's protective effect, with quantitative analysis of the changes in mutant opsin localization to the OS versus retention at ER or degradation at proteosome.

In *Rpgrip1^−^^/^^−^* and *rd10* mice, augmented HSP70 expression did not demonstrate a protective effect; instead, we observed an accelerated degeneration in the *rd10* retina and no apparent impact in the *Rpgrip1^−^^/^^−^* retina. RPGRIP1 functions as a docking site for ciliary proteins at the base of the connecting cilium and likely facilitates trafficking of nascent proteins from the IS into the OS.^38^^,^^55^^–^^57^ With loss of RPGRIP1, RPGR fails to anchor at the base of the connecting cilium and the proper transport and localization of rod and cone opsin are affected.^38^ Enhancing the chaperone system does not rescue the phenotype, and no protective effect was observed. The *rd10* mice carry a missense mutation in the gene encoding β-subunit of cGMP phosphodiesterase 6 (*Pde6b*).^45^ The *rd10* allele produces a hypomorphic PDE6B that is partially defective in assembling into the PDE6αβγ2 holoenzyme with decreased transport to the OS, leading to higher free cGMP, increased channel opening and Ca^2+^ influx, and rapid photoreceptor death.^58^^–^^60^ We were intrigued by the accelerated photoreceptor degeneration in *rd10* retina by HSP70 overexpression. The mutant PDE6B is expressed at low levels in *rd10* retina, exhibits residual catalytic activity, and is partially mislocalized, consistent with a misfolding defect.^58^ Increased expression of HSP70 would enhance the quality control mechanisms, which target misfolded proteins,^61^ as evident by decreased staining of PDE6B in *rd10;TgHspa1a+* mice (Fig. 7). Photoreceptor specific chaperone AIPL1^27,28^ directly interacts with HSP70 and HSP90,^25^^,^^62^ forming a chaperone heterocomplex that regulates biosynthesis, stability, and translocation of PDE6 holoenzyme.^26^ HSP70 and HSP90 may compete for binding to the client protein PDE6B. We suggest that overexpression of one arm of the chaperone heterocomplex may lead to imbalance of the system, enhancing PDE6B degradation and accelerating photoreceptor degeneration. Thus augmenting chaperone activity might have limited or no effect when dealing with protein-null defects such as *Rpgrip1^−^^/^^−^*, and overstimulating the chaperone machinery may accelerate degeneration in specific cases such as *rd10* mice.

It is unclear why improved rod survival in *RHOT17M* mice was not accompanied by a corresponding improvement in dark-adapted ERG amplitudes. HSP70 may assist mutant rhodopsin folding and/or trafficking to the correct subcellular compartment (i.e., the OS). Although likely relieving ER stress and thereby promoting cell survival, increased mutant rhodopsin in the OS would interfere with phototransduction. Misfolded mutant rhodopsin that fails to fully regenerate with the chromophore can stimulate phototransduction via transducin activation in the absence of light and reduce rods sensitivity because full dark adaptation is not achievable, as if they are constantly exposed to light.^49^ We hypothesize that increased trafficking of mutant rhodopsin to the OS may at least in part account for the lack of ERG improvement in this model. Surprisingly, we observed drastically reduced photopic a- and b-wave amplitude in *rd10* mice at P30 and significantly reduced photopic b-wave amplitude in *Rpgrip1^−^^/^^−^* mice at P40 with augmented HSP70 expression, suggesting compromised cone function or survival. As the *TgHspa1a* transgene has no deleterious effect on cone function and survival in wild-type background, a likely scenario in *rd10* retina might be that accelerated rod decline secondarily impacted cone viability.

## Conclusions

Members of HSP70 family usually work together with HSP40 (DNAJ), HSP90, and HSP110 family co-chaperones, forming a large and complex chaperone network in maintaining cellular proteostasis. DNAJs, which regulate HSP70's function as co-chaperones, play an important role in suppressing disease-causing protein aggregations.^63^ DNAJB6b and DNAJB8 are shown to be superior suppressors than HSP70 in treating aggregates associated with polyglutamine expansion.^64^ Thus HSP70 may not be the only or best candidate for therapies targeting the chaperone system. We had previously demonstrated retinal expression of the small HSP α-crystallin, traditionally believed to localize in the ocular lens.^65^ Expression of α-crystallin is elevated in diseased retina where it may play a protective role.^66^^,^^67^ In some cases, augmentation of HSP function can be a double-edged sword, and augmentation of different HSPs might result in unintended consequences. A member of the 90 KDa family, HSP90α, predominantly expressed in the retina and brain,^68^ has also been targeted for treating retinal degeneration.^69^ Although inhibition of HSP90 protected dying photoreceptors in vivo,^31^^,^^70^ prolonged HSP90 inhibition led to photoreceptor death in beagle dogs,^71^ and HSP90α-deficient mice displayed retinal degeneration.^68^ Given that HSP70 augmentation in our studies showed distinct impact on degenerating photoreceptors from different mutants, we suggest that a better understanding of the chaperone machinery and especially HSPs in degenerating retinas is required and that various chaperone candidates should be considered when developing treatment paradigms focused on photoreceptor protein homeostasis.
